# Tailoring Risperidone-Loaded Glycethosomal In Situ Gels Using Box–Behnken Design for Treatment of Schizophrenia-Induced Rats via Intranasal Route

**DOI:** 10.3390/pharmaceutics15112521

**Published:** 2023-10-24

**Authors:** Marwa H. Abdallah, Hemat El-Sayed El-Horany, Hanan M. El-Nahas, Tarek M. Ibrahim

**Affiliations:** 1 Department of Pharmaceutics, College of Pharmacy, University of Ha’il, Ha’il 81442, Saudi Arabia; 2 Department of Pharmaceutics, Faculty of Pharmacy, Zagazig University, Zagazig 44519, Egypt; hananelnahas@gmail.com (H.M.E.-N.); tarekmetwally333@gmail.com (T.M.I.); 3 Department of Biochemistry, College of Medicine, University of Ha’il, Ha’il 81442, Saudi Arabia; h.elhorany@uoh.edu.sa; 4 Department of Medical Biochemistry, Faculty of Medicine, Tanta University, Tanta 31511, Egypt

**Keywords:** risperidone, glycethosomal vesicles, in situ intranasal gel, ex vivo permeation, pharmacokinetic study, ELISA assay

## Abstract

Schizophrenic patients often face challenges with adherence to oral regimens. The study aimed to highlight the potentiality of intranasal ethanol/glycerin-containing lipid-nanovesicles (glycethosomes) incorporated into in situ gels for sustaining anti-psychotic risperidone (RS) release. The Box–Behnken Design (BBD) was followed for in vitro characterization. Glycethosomal-based in situ gels were examined by physical, ex vivo, and in vivo investigations. The ethanol impact on minimizing the vesicle size (VS) and enhancing the zeta potential (ZP) and entrapment efficiency (EE%) of nanovesicles was observed. Glycerin displayed positive action on increasing VS and ZP of nanovesicles, but reduced their EE%. After incorporation into various mucoadhesive agent-enriched poloxamer 407 (P407) in situ gels, the optimized gel containing 20% P407 and 1% hydroxypropyl methyl cellulose-K4M (HPMC-K4M) at a 4:1 gel/glycethosomes ratio showed low viscosity and high spreadability with acceptable pH, gel strength, and mucoadhesive strength ranges. The ethanol/glycerin mixture demonstrated a desirable ex vivo skin permeability of RS through the nasal mucosa. By pharmacokinetic analysis, the optimized gel showed eight-fold and three-fold greater increases in RS bioavailability than the control gel and marketed tablet, respectively. Following biochemical assessments of schizophrenia-induced rats, the optimized gel boosted the neuroprotective, anti-oxidant, and anti-inflammatory action of RS in comparison to other tested preparations. Collectively, the intranasal RS-loaded glycethosomal gel offered a potential substitute to oral therapy for schizophrenic patients.

## 1. Introduction

Schizophrenia is a persistent and severe psychiatric condition characterized by the presence of positive symptoms (such as hallucinations and delusions), negative symptoms (including disruptions in normal behavior and emotion), and cognitive symptoms (manifesting as impairments in memory and attention) [1]. The onset of symptoms associated with schizophrenia typically occurs during the maturity and persists throughout the individual’s lifespan [2]. The aforementioned symptoms can be effectively controlled with the use of anti-psychotic medications, particularly atypical anti-psychotic ones [2]. Risperidone (RS) is one of these atypical anti-psychotic medicines. It is a derivative of benzisoxazole and is approved for the management of both chronic and acute schizophrenia. The principal mechanism of RS action is the reduction of serotonergic (5-HT_2A_) and dopaminergic (D_2_) pathway activities inside the brain [3]. Due to its moderate affinity for D_2_ receptors and high affinity for 5-HT_2A_ receptors, it can lessen the negative and positive signs of schizophrenia, respectively, while potentially reducing the extrapyramidal adverse effects [4].

Lipid-based vesicular drug delivery systems have garnered significant attention as promising technologies for enhancing the absorption of drugs through the skin and mucosal membranes. This approach may have numerous benefits, including overcoming the drug degradation in the gastrointestinal tract, circumventing the hepatic first pass metabolism, and enhancing the compliance among patients [5]. Liposomes are vesicles composed of phospholipids (PLs), which make them the initial generation of vesicular carriers based on lipids. The lipid-based drug delivery systems have the ability to encapsulate both hydrophilic and lipophilic medicinal agents. These systems consist of one or more lipid bilayers that surround an aqueous region. In recent years, there have been developments in modified drug delivery systems, such as ethosomes, transfersomes, glycerosomes, and glycethosomes. These advancements have been made with the objective of enhancing the ability of medicinal agents to penetrate the biological membranes [6]. These innovative systems, also known as elastic or ultra-deformable liposomes, have undergone significant investigations for drug delivery to the mucosal membrane [7].

Glycerosomes are recently developed PL-based vesicular systems that incorporate glycerol into the aqueous phase [8]. The utilization of these systems has been examined for delivering medicinal agents through the topical, transdermal, and intranasal routes [5,9]. Additionally, there has been a recent focus on investigating their potential to deliver drugs by inhalation [10]. The presence of glycerol in significant quantities enhances the fluidity of the lipid bilayer in liposomes, hence enhancing the vesicular penetration and absorption of drugs [5]. Glycethosomes, as novel vesicular systems, are a modified version of glycerosomes containing a reasonably specific quantity of ethanol. The vesicles exhibit a notable level of deformability, allowing them to penetrate effectively through the intact skin and mucosal membranes.

The term “in situ gel systems” pertains to polymer solutions that can be supplied in liquid form and afterwards undergo a phase transition to become semisolid gels when exposed to a physiological stimuli [11]. There are several mechanisms that might result in the development of in situ gels, including pH variation, ionic cross-linkage, and temperature variation [11]. Their fluid-like fluidity before coming into contact with the nasal mucosa can make them easier to administer and enable for a precise drug dosing. Furthermore, the extended duration of their presence at the site of application can lead to an augmentation in the drug penetration across the nasal mucosa, hence enhancing its bioavailability [12]. On another side, utilizing mixtures of gelling agents is interestingly followed to acquire more benefits to the in situ gel products after observing their behavior [13,14]. These mixtures can offer new points of view about the thermo-sensitive delivery systems, for example, positively affecting their gel strength and elasticity, improving their physical properties, controlling the drug release, and facilitating the dosage administration [15]. Therefore, choosing appropriate polymer mixtures at specific concentrations and adjusted conditions would develop the process and result in desirable and well-controlled delivery systems.

The utilization of intranasal drug administration has received a great amount of interest within the drug delivery field due to its notable advantages. The nasal route has several advantages, such as circumventing the medications’ pre-systemic metabolism and providing a rapid beginning of effect in comparison to the alternative routes like oral and transdermal administration. This is due to the abundant blood supply in the nasal mucosa. Moreover, the nasal route represents an effective and non-invasive method for administering drugs by patients [16].

To the best of our knowledge, there have been no studies on the utilization of glycethosomal in situ gel to improve the bioavailability of RS. Furthermore, research on the potential of glycethosomal in situ gel to improve the transmucosal penetration of the drugs via the nasal cavity is lacking. Thus, the current investigation focuses on the design and formulation of a glycethosomal in situ gel loaded with RS for studying its action on managing the schizophrenia disease induced in rats followed by the pharmacokinetic and biochemical assessments. The target is to improve the RS bioavailability by the intranasal administration, enhance the compliance of schizophrenic patients, and minimize the systemic toxic effects.

## 2. Materials and Methods

### 2.1. Materials

Risperidone (RS) was acquired from Corey Organics Co., Visakhapatnam, India. Epikuron 200 phospholipid was kindly supplied from Cargill Texturizing Solutions, Deutschland GmbH & Co., Hamburg, Germany. Cholesterol was obtained from BDH Chemicals Ltd., Poole, UK. Absolute ethanol, glycerin, propylene glycol (PG), isopropyl alcohol, disodium hydrogen orthophosphate, and potassium dihydrogen orthophosphate were procured from El-Nasr Pharmaceutical Chemicals, Cairo, Egypt. Hydroxypropyl methyl cellulose-K4M (HPMC-K4M), carbopol 940, and polyvinylpyrrolidone K30 (PVP K30) were obtained from Egyptian International Pharmaceutical Industries, 10th of Ramadan, Egypt. Poloxamer 407 (P407) was obtained from Sigma, St. Louis, MO, USA. Sodium azide was purchased from Universal Fine Chemical Pvt Ltd., Mumbai, India. Acetonitrile was supplied from Fisher Scientific, Leicestershire, UK. Triethylamine was purchased from Loba Chemie, Mumbai, India. Ketamine was purchased from the local pharmacy.

### 2.2. Statistical Design of RS-Loaded Lipid-Based Nanovesicles

This investigation was designed using a Box–Behnken Design (BBD) to address the effect of different variables of formulation on the characteristics of RS-loaded vesicular systems using Design Expert^®^ software (Ver. 11, Stat-Ease, Minneapolis, MN, USA) [17]. The independent variables (factors) were the amount of PL (A), which ranged from 200 mg to 400 mg, ethanol concentration (B), which lay between 0% *v*/*v* and 20% *v*/*v*, and glycerin concentration (C), which ranged from 0% *v*/*v* to 20% *v*/*v*. This resulted in 17 experimental runs. The dependent variables (responses) were vesicle size (VS; R_1_), zeta potential (ZP; R_2_), and entrapment efficiency (EE%; R_3_). Table 1 shows the independent variables (low and high levels) and the required goals for the dependent variables. Table 2 shows the composition and the measured responses of the RS-loaded formulations.

The analysis of variance (ANOVA) was followed for analyzing the significance of effect of each factor on the responses. The following statistical parameters were measured to assess the fitness of the data, for example, multiple correlation coefficient (R^2^) and adjusted/predicted R^2^ values. The adequate precision values were noticed. Additionally, the plots of predicted versus actual values, normal plots of residuals, and graphs of residual versus experimental runs were tested for estimating the adequacy of the studied models.

### 2.3. Preparation of Lipid-Based Nanovesicles Loaded with RS

A measured amount of RS (10 mg) was dissolved in a mixture of ethanol, glycerin, and PG at various ratios as given in Table 2. PL and cholesterol (20 mg) were swelled in distilled water using a hot plate stirrer (AREC F20500010, VELP Scientifica, Brianza, Italy) set at 60 °C and 1500 rpm. The drug solution was thereafter added dropwise to the lipid mixture with a continuous stirring for 1 h at 60 °C and 1500 rpm till formation of dispersions having final volumes of 5 mL. The resulting lipid dispersions were subjected to four cycles of 1 min of sonication in an ice bath using a probe sonicator (GE 50, Scientific Engineering Inc., Woodbridge, VA, USA) and then exposed to refrigeration overnight for further studies [18].

### 2.4. Characterization of RS-Loaded Lipid-Based Nanovesicles

#### 2.4.1. Measurement of VS and ZP

The VS and ZP values of the prepared formulations were determined after dilution to 100 times with distilled water using Zetasizer instrument (Nano–ZS90, Malvern Instruments Ltd., Malvern, UK) employing the dynamic light scattering approach [19]. The experiment was performed in triplicate and the results were mentioned as mean values ± standard deviation (S.D).

#### 2.4.2. Measurement of EE%

The amounts of drug encapsulated in the vesicles were measured by centrifuging the prepared formulations for 1 h at 18,000 rpm and 4 °C (Z 300 K, Hermle Labortechnik GmbH, Wehingen, Germany). The supernatants were collected and used for assessing the drug contents spectrophotometrically at λ_max_ 278 nm (Genesys 10S UV-VIS, Thermo Spectronic, New York, NY, USA) [20]. The EE% values were measured three times and calculated following this equation:

(1) $$EE\%=\frac{Original\;amount\;of\;drug-Free\;amount\;of\;drug}{Original\;amount\;of\;drug}\times 100$$

### 2.5. Optimization of RS-Loaded Lipid-Based Nanovesicles

As suggested by the BBD, an optimized formulation was selected according to its desirability value. Such value was acceptable when being close to one. The relationship between the predicted and observed responses of the optimized formulation was examined.

### 2.6. Characterization of Optimum RS-Loaded Lipid-Based Nanovesicles

#### 2.6.1. Transmission Electron Microscopy (TEM) Study

A droplet of the diluted dispersion was allowed to undergo absorption on a grid coated with carbon for a duration of 2 min. Subsequently, the droplet was covered with a droplet of 1% aqueous solution of phosphotungstic acid and observed using a transmission electron microscope (JEM-2100, JEOL, Tokyo, Japan) operating at an accelerating voltage of 100 kV.

#### 2.6.2. Differential Scanning Calorimetry (DSC)

The DSC thermograms of pure PS powder, PL, cholesterol, and optimized RS-loaded glycethosomal formulation were recorded on a calorimeter (DSC-60, Shimadzu, Kyoto, Japan) computerized with an operating software system. By a careful sampling, small amounts of samples were placed into small aluminum pans. The pans were tightly sealed and heated from 0 °C to 200 °C at a fixed rate of 10 °C/min. The heating was maintained under a nitrogen flow of 20 mL/min.

#### 2.6.3. Fourier Transform Infrared (FTIR) Spectroscopy

The FTIR spectra of pure RS powder, PL, cholesterol, and optimized RS-loaded glycethosomal formulation were recorded for determining the compatibility of RS and excipients using a Perkin-Elmer 1600 FTIR spectrophotometer with a scanning range of 4000–400 cm^−1^.

#### 2.6.4. In Vitro Release Study

Using cellophane membrane dialysis tubing (molecular weight cut-off 12,000–14,000 Da), the in vitro release of RS from the obtained optimized formulation was carried out [21]. To ensure the complete wetting of the membranes, they were hydrated in the receptor compartment (phosphate-buffered saline of pH 6.4) overnight before dialysis. The dialysis bags were filled with the formulation (equivalent to 2 mg RS) and sealed from the both ends. Then, the bags were placed in firmly closed bottles filled with 20 mL of receptor compartment and placed in a water bath shaker (SW-20C, Julabo Labortechnik GmbH, Seelbach, Germany) at 34 ± 1 °C. Samples of one milliliter were extracted at predetermined time intervals (0.5, 1, 2, 3, 4, 5, 6, 7, 8, and 24 h) and afterwards subjected to filtration using a 0.22 μm nylon syringe filter. To ensure a consistent volume, an equal amount of fresh buffer was introduced into the receptor compartment. The spectrophotometric method was employed to assess the mean values of drug content at 278 nm. The mean values (n = 3) were then compared to the drug content values of the pure drug.

### 2.7. Preliminary Study for Preparation of Intranasal RS-Loaded Glycethosomal In Situ Gels

The in situ gels were prepared by the cold method described by Choi et al. [22] and Schmolka [23]. Briefly, different concentrations of P407 (17–20% *w*/*v*) were added slowly to cold distilled water with simultaneous and constant agitation for 15 min, then the formulations were refrigerated at 4 °C until transparent liquids were obtained [24]. After selecting a specific concentration of P407 at which the polymeric solution converted to a gel at the solution-to-gel temperature (T_sol-gel_) of 34 °C resembling the nasal temperature [25], such P407 concentration was used for further evaluation.

Different mucoadhesive polymers (carbopol 940, HPMC-K4M, or PVP K30) at different concentrations were added slowly with agitation to the selected P407 polymeric solution and cooling using an ice bath. The samples underwent examination to assess the gelation time and gel strength parameters after adding the optimized glycethosomal formulation to the generated polymeric gels at different gel/liquid glycethosomes ratios. The selection of optimal in situ glycethosomal gel formulation was followed after examining the pH, viscosity, spreadability, mucoadhesive strength, and in vitro release studies.

### 2.8. Characterization of RS-Loaded Glycethosomal In Situ Gels

#### 2.8.1. Gelation Time Determination

By the gel inversion method, approximately 5 g of the generated preparations were placed in glass vials, and then transferred to a thermobalanced water bath at a temperature of 34 °C. The time required for transition of the generated gels from liquid to gel forms was estimated.

#### 2.8.2. Gel Strength Assessment

The gel strength was also evaluated by first adding a particular weight (3.5 g) on the top of each gel sample (5 g), which was placed in a graduated measure. A timer was used to record how long the weight took to pierce the sample for 3 cm. For gels, the time recorded from 25 to 50 s was optimum.

#### 2.8.3. pH Evaluation

The pH values were determined by dipping the glass electrode of a digital pH meter (5005, Jenco Instruments, San Diego, CA, USA) into a sufficient amount of each generated formulation at room temperature.

#### 2.8.4. Viscosity Evaluation

The viscosity of the in situ gels was estimated be immersing the spindle of the viscometer (Visco Star-R, Fungilab S.A., Barcelona, Spain) within the generated gels and rotating at 10 rpm.

#### 2.8.5. Spreadability Evaluation

The values of gel spreadability were calculated by spreading 1 g of each gel sample in a circle with a diameter of 1 cm between two opposite glass plates. A particular weight (0.5 Kg) was then applied over the plates. The sizes of circles were varied because of the gels spreading and these variations were noted.

#### 2.8.6. Mucoadhesive Strength Measurement

Fresh sheep nasal mucosa and modified physical balance were used to determine the mucoadhesive ability of the investigated formulations (Figure S1). The technique involved measuring how much power was needed to separate the gel formulation from the nasal membrane. An empty plastic cup was used in place of the right pan of the balance. Two glass vials joined base to base were used in place of the left pan of the balance. Two mucosal pieces that had been moistened with phosphate-buffered saline (pH 6.4) were added to the base of each vial and the tested gel formulations were placed between the two mucosal parts. To ensure the close contact of the two glass vials and the release of the trapped air between the two vials, a specified weight was applied to the upper vial for two minutes. Water was slowly and steadily poured into the plastic cup until both vials were separated [26]. The following formula was used to calculate the mucoadhesive strength:

(2) $$Strengh\;of\;mucosdhesion=\frac{m\times g}{A}$$

where g is the acceleration of gravity (980 cm/s^2^), m is the weight of the additional water (gram), and A is the area of the exposed mucosa (cm^2^).

#### 2.8.7. In Vitro Release Study

The release of RS from the in situ gel samples was carried out using the cellophane membrane dialysis tubing method following the procedures shown in Section 2.6.4.

All previous in vitro tests performed on the RS-loaded glycethosomal in situ gels were carried out in triplicate and their measurements were expressed as mean values ± S.D.

After examining the aforementioned parameters, a RS-loaded glycethosomal in situ gel was selected as an optimized formulation.

### 2.9. Characterization of Optimized RS-Loaded Glycethosomal In Situ Gel Formulation

#### 2.9.1. Ex Vivo Drug Permeation Study

A fresh nasal tissue extracted from the sheep nasal cavity was used to study the ex vivo RS penetration characteristics. The well cleaned mucosal membrane was attached to a diffusion cell with a 2.54 cm^2^ penetration area. The donor compartment was filled with the optimized glycethosomal in situ gel or the control gel, and both were equivalent to 2 mg RS. Samples (1 mL) were taken out of the acceptor compartment, which contained 20 mL of phosphate-buffered saline (pH 6.4) with 0.02% sodium azide as a preservative, at specified intervals and appropriately quantified spectrophotometrically at 278 nm. The cumulative amounts of RS penetrated per unit area (μg/cm^2^) versus time were plotted to produce the slope of the linear component of the curve, which allowed the measurement of the drug flux at steady state (J_ss_). By dividing the flux by the initial quantity of the drug employed, the permeability coefficient (K_p_) was calculated. The enhancement ratio (E_r_) of the investigated formula to the control formula was also calculated (J_ss_ formula/J_ss_ control). The values were demonstrated as means ± S.D (n = 3).

#### 2.9.2. Histopathological Investigation

Three nasal mucosa samples that were all of the same size were checked. Saline was used to treat the first piece (negative control). The second piece served as a positive control and was given treatment with a strong mucociliary toxin (isopropyl alcohol). In comparison, the mucosal piece remaining after the ex vivo permeation study that was treated with the optimized gel formulation was histopathologically examined. The mucosal membranes were washed with phosphate-buffered saline (pH 6.4) and then left in 10% neutral formalin overnight. After that, they were vertically sliced and dried using ethanol. These mucosal sections were mounted in paraffin blocks and, by using a microtome, thin sections were cut and stained with hematoxylin and eosin. They were looked at under a microscope at a 400× magnification power [27].

#### 2.9.3. In Vivo Evaluation

##### Animals

The study included adult male albino rats with weights of 250 g. The rats utilized in this study were purchased from the animal breeding unit at Zagazig University, located in Egypt. The animals were housed under standard laboratory conditions with a controlled environment of room temperature and a 12 h light–dark cycle. They were provided with free access to both food and water. The rats had a period of acclimatization for a minimum of one week prior to commencing the studies. The animals were subjected to treatment in accordance with the criteria established by the Institutional Animal Care and Use Committee (IACUC) of the Faculty of Pharmacy at Zagazig University. The study was conducted under the approval number ZU-IACUC/3/F/357/2022.

The optimized intranasal glycethosomal in situ gel formulation, intranasal control in situ gel, and oral marketed tablet (Riscure^®^, Global Napi Pharmaceuticals, 6th of October, Egypt) were modified to attain 0.3 mg RS per kilogram of rats’ body weight [28]. The animals were divided randomly into five groups (n = 6). Group I: rats kept as control and fed a normal diet at corresponding times. Group II: rats induced with schizophrenia by injection of ketamine and kept as negative control. Group III: schizophrenic animals received the intranasal control in situ gel. Group IV: schizophrenic animals received the oral marketed tablet. Group V: schizophrenic animals received the selected intranasal RS-loaded glycethosomal in situ gel formulation.

##### Induction of Schizophrenia in Rats

In rats, ketamine can have psychomimetic effects when used in sub-anesthetic doses. Consequently, the onset of schizophrenia was generated in rats with the administration of ketamine through the intraperitoneal injection on a daily basis for a period of 14 consecutive days with each injection containing a dosage equivalent to 30 mg/kg [29]. Animal group II received ketamine alone (30 mg/kg) daily for 14 days. Animal groups III, IV, and V received ketamine alone (30 mg/kg) daily for the first 7 days and then received both ketamine (30 mg/kg) and the studied vehicles daily for the second 7 days with a 30 min interval between the two treatments.

##### Calculation of Pharmacokinetic Parameters

The subsequent determination of pharmacokinetic parameters was performed after administration of studied formulations. At the last day of the study, samples of blood were collected from the lateral tail vein of animal groups III, IV, and V using heparinized tubes at various time intervals including 0, 1, 2, 3, 4, 6, 8, and 24 h. The samples of blood were subjected to centrifugation for a duration of 10 min at 3000 rpm, resulting in clear plasma separation. The plasma samples were stored at a temperature of −20 °C until they were ready for analysis.

In order to facilitate the high-performance liquid chromatography (HPLC) analysis (2690 Waters, Milford, Godalming, UK), the plasma samples that had been separated were allowed to thaw at an ambient temperature. Subsequently, 400 μL of each sample was combined with 1 mL of acetonitrile and subjected to vortexing for a duration of 2 min using a vortex mixer (Purimix, Cryste-Novapro, Bucheon, Republic of Korea). The samples were thereafter subjected to centrifugation at a speed of 15,000 rpm for duration of 10 min at 4 °C using a refrigerated centrifuge. The supernatants were subjected to evaporation using a nitrogen stream. They were reconstituted with 120 μL of the mobile phase and filtered using a 0.22 μm syringe filter. The mobile phase utilized in the experiment was composed of a solution containing 0.05 M monobasic potassium phosphate with a pH of 3.5 combined with acetonitrile at a ratio of 65:35% *v*/*v*. Additionally, triethylamine was included in the mobile phase at a concentration of 0.3% *v*/*v*. The flow rate of the mobile phase during the experiment was maintained at 1 mL/min. The 100 μL samples were introduced into a C18 thermal column with a particle size of 5 μm and dimensions of 4.6 × 250 mm while maintaining the ambient temperature. The detection wavelength employed in this study was 278 nm utilizing a photodiode array detector (996, Waters, Milford, CT, USA). The mobile phase underwent degassing through the process of sonication.

The pharmacokinetic calculations were performed using the non-compartmental technique with the assistance of the add-in PKsolver tool by Microsoft Excel, version 2. The values of maximum plasma concentration (C_max_) and the time taken to achieve this concentration (t_max_) were obtained directly from the profiles of plasma concentration with time. The determination of the apparent elimination rate constant (K_el_) involved the analysis of the terminal slope observed in the plasma concentration–time curves. The area under the first moment curve (AUMC_0-∞_) and the area under the plasma concentration–time curve (AUC_0-t_) were both calculated using the trapezoidal method. The calculations were conducted in order to identify the elimination half-life (t_1/2_), the area under the curve from time zero to infinity (AUC _0-∞_), mean residence time (MRT), and relative bioavailability. The pharmacokinetic values were stated as means ± S.D (n = 6).

##### Enzyme-Linked Immunosorbent Assay (ELISA) Analysis

The animals were subjected to euthanasia, following which their hippocampus tissues were carefully excised and promptly cleaned in a solution of ice-cold saline. The hippocampus tissues were ultimately homogenized in a saline solution and afterwards utilized for subsequent biochemical assessment [30]. Commercially available kits of ELISA were utilized to determine the levels of dopamine (MBS7214676, MyBiosource, Inc., San Diego, CA, USA), serotonin (MBS725497, MyBiosource, Inc., San Diego, CA, USA), malondialdehyde (MDA) (LS-F28018 LifeSpan Biosciences, Inc., Seattle, WA, USA), tumor necrosis factor-α (TNF-α) (MBS355371, MyBiosource, Inc., San Diego, CA, USA), and brain derived neurotrophic factor (BDNF) (MBS355345, MyBiosource, Inc., San Diego, CA, USA) in the hippocampus of different treated animal groups. The ELISA assays were conducted in strict adherence to the instructions provided by the manufacturer.

### 2.10. Statistical Analysis

The parameters were subjected to statistical analysis using ANOVA followed by Tukey’s post hoc test. The program utilized for the analysis was GraphPad Prism^®^ (San Diego, CA, USA) ver. 5.00.

## 3. Results and Discussion

### 3.1. Statistical Analysis by BBD

As per the data of Table S1, the ANOVA findings showed that the models had a statistical significance as represented by their high F-values and low *p*-values (<0.0001) indicating a low chance of noise exhibited by these F-values. The significance of the studied terms of the models was observed in relation to their *p*-values. In addition, the R^2^ values, measuring the variation amount around the means given by the models, were close to one, hence clarifying the satisfactory correlation between the actual and predicted values (Figure S2) [31]. Additionally, there was a good consistence between the adjusted and predicted R^2^ values. This could be indicated by the low difference between them (<0.2) as suggested by the design software. The adequate signal/noise ratios were also observed by the values of the adequate precision (>4; as proposed by the design software) [32].

Furthermore, the diagnostic plots of the three models were also analyzed. The normal plots of residuals represented that the differences between the actual and predicted values, recognized as residuals, were closely fitted on straight lines and normally distributed (Figure S3). The plots of externally studentized residuals versus runs displayed that the points were present randomly between the control limits, therefore proving the low chances of errors and the marked desirability and fitness of models (Figure S4) [33].

#### 3.1.1. Effect of Independent Factors on VS (R_1_)

**Figure 1:**
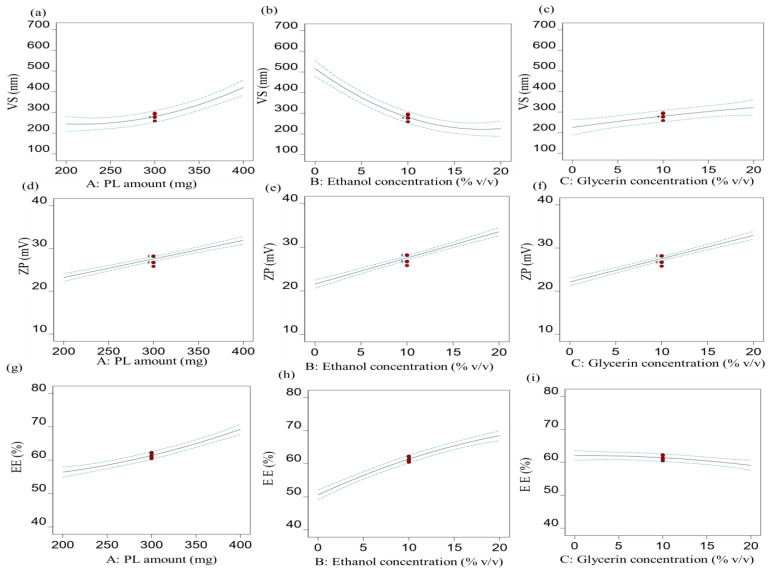
BBD plots demonstrating the effect of (**a**) variable A on R_1_, (**b**) variable B on R_1_, (**c**) variable C on R_1_, (**d**) variable A on R_2_, (**e**) variable B on R_2_, (**f**) variable C on R_2_, (**g**) variable A on R_3_, (**h**) variable B on R_3_, and (**i**) variable C on R_3_.

As summarized in Table 2, the mean VS of different nanovesicles fluctuated from 163.10 ± 7.45 nm to 646.80 ± 31.65 nm and was affected by various formulation variables. As shown in Figure 1a, there was a direct relationship between the PL amount and VS. The mean VS of the prepared vesicles (glycethosomes) with 200 mg PL (F10; 163.10 ± 7.45 nm) was significantly smaller than that prepared with 400 mg PL (F3; 410.50 ± 14.08 nm). The high VS values could be attributable to the high rigidity of the prepared vesicles and the formation of highly thickened structures caused by the increment of PL amounts [34].

It was noted that elevating the ethanol concentration led to a reduction in the size of the prepared nanovesicles (Figure 1b). The mean VS value of the formulation (F3, glycethosomes) containing 20% ethanol was 410.50 ± 14.08 and smaller than the glycerosomal formulation (F1; 646.80 ± 31.65), which had no ethanol.

Several investigations indicated that the elevated concentration of ethanol might induce the interpenetration of the ethanol hydrocarbon chain, potentially resulting in a decrease in the thickness of the vesicular membrane and consequently reducing the VS. Moreover, addition of ethanol led to a reduction in the VS due to its steric stabilizing effect on the formulation leading in a subsequent reduction in the VS [35]. In addition, ethanol could function as a source of negative charges on the surface of nanovesicles (ethosomes and glycethosomes). This property would prevent the vesicular systems from aggregating by means of electrostatic repulsion. Furthermore, it had been shown that the stabilizing properties of ethanol could lead to a subsequent reduction in size [35,36].

At fixed PL amount (A) and ethanol concentration (B), increasing the glycerin concentration (C), resulted in a concomitant increase in the VS as shown in Figure 1c. Nanovesicles prepared with 0% glycerin concentration (F11; ethosomes) showed a smaller VS (180.90 ± 16.73 nm) compared to the glycethosomal nanovesicles prepared with 20% glycerin concentration (F4; 328.90 ± 20.11 nm). The increment of glycerin concentration led to increased VS values probably due to the sticky texture of glycerin [9,37]. Additionally, the glycerin and ethanol combination could lose the packing of the membranes of the prepared vesicles, hence decreasing the bilayer curvature and producing large-sized vesicles [9]. The equation attained for this quadratic model was

(3) $$R_{1}=280.54+88.15A-145.51B+48.11C+18.58AB-14.48AC-15.90BC+52.13A^{2}-90.86B^{2}-6.59C^{2}$$

In coordination with the data of Table S1, the equation showed that the VS of the nanovesicles was positively influenced by the factors A and C, but negatively influenced by the factor B in a significant manner. The quadratic terms of factors A and B displayed significant effects on the R_1_ response as shown by their low *p* values, while the other terms were non-significant.

#### 3.1.2. Effect of Independent Factors on ZP (R_2_)

Figure 1d–f show that the absolute values of the ZP increased as the concentrations of PL, ethanol, and glycerol increased. The ZP of all prepared RS-loaded nanovesicles had negative values ranging from −15.92 ± 1.31 mV to −39.25 ± 0.42 mV (Table 2). The negative charges could produce a repulsion force between the prepared vesicles and protect them from agglomeration or instability [38].

The significant positive effect of raising the PL amounts on increasing the ZP values was observed. This might be ascribed to the ability of PLs to increase the rigidity of the vesicular layers and minimize the likelihood of the fusion of lipidic vesicles, hence producing evenly distributed and stable vesicles [34]. The increased ZP values resulted by high ethanol concentration might be attributed to the fact that ethanol could provide a concentration-dependent negative charge to the polar heads of PLs that would develop an electrostatic repulsion and reduce the vesicles aggregation [39].

Regarding factor C, the absolute levels of ZP were increased by raising the glycerin concentration. This phenomenon could be elucidated by the unique interaction between glycerin and the polar heads of PLs resulting in a reorientation of molecules, ultimately impacting the overall surface charge of the system [40]. The equation of this linear model was determined as shown below:

(4) $$R_{2}=27.55+4.34A+5.99B+5.40C$$

The equation showed that the ZP of the nanovesicles was positively and significantly influenced by the factors A, B, and C. The model was linear and there were no interaction effects recorded (Table S1).

#### 3.1.3. Effect of Independent Factors on EE% (R_3_)

According to the data presented in Table 2, the EE% values exhibited a range from 47.22 ± 1.95% to 78.16 ± 1.68%. The PL concentration significantly affected the EE% of the vesicles. It was revealed that an increase in EE% was observed with an increase in the PL concentration (Figure 1g). The formulation (F3), which contained 400 mg of PL, demonstrated an EE% of 78.16 ± 1.68% compared to that of the formulation (F10) containing 200 mg of PL (60.58 ± 0.57%). Similar findings were shown when comparing the ethosomal formulation (F6) with 400 mg PL (70.30 ± 1.81%) and formulation (F11) with 200 mg PL (44.43 ± 1.33%). The increase in the EE% with an increase in the PL concentration could be related to the lipophilicity of RS, since the lipophilic medication would find a high lipid surface area to be encapsulated in the lipoid phase of the vesicles [41].

As illustrated in Figure 1h, ethanol had a positive impact on the entrapment of RS in the lipid-based nanovesicles. There was an increase in the EE% that was observed by increasing the ethanol concentration. The EE% of formulation (F1) with 0% ethanol was 57.86 ± 0.87%, but for formulation (F3) with 20% ethanol, it was 78.16 ± 1.68%. The EE% was shown to be 47.62 ± 1.65% for formulation (F8) with 0% ethanol and 67.20 ± 1.15% for formulation (F2) with 20% ethanol. Similar findings were observed between formulations F13 and F10 (Table 2). The improvement in the aqueous solubility of RS was achieved by utilizing high concentrations of ethanol. This could be due to its co-solvent effect. Therefore, more drug amount could be accommodated in the aqueous core of the vesicles [42,43].

Glycerin had a negative effect on the EE%. This value reduced as the glycerin concentration increased (Figure 1i). The EE% of formulation (F6; ethosomes with 0% glycerin concentration) was 70.30 ± 1.81%, while the formulation (F14; glycethosomes with glycerin 20%) had an EE% of 65.23 ± 1.81%. In addition, similar findings were observed between formulation (F15; 0% glycerin) (EE% of 50.83 ± 0.89%) and formulation (F8; 20% glycerin) (EE% of 47.62 ± 1.65%). These results are consistent with those obtained by Younis and Habib who demonstrated that glycerin had a negative impact on the encapsulation of sertaconazole nitrate-loaded glycerosomes [20]. It was also reported that higher glycerin concentrations in the aqueous phase could raise the solubility of drug in the intervesicle medium of glycerin causing a possible leakage of drug [5].

The equation for this quadratic model was determined as

(5) $$R_{3}=61.39+6.42A+8.94B-1.48C+1.73AB-0.85AC+0.33BC+1.43A^{2}-1.87B^{2}-0.84C^{2}$$

The polynomial equation showed that the EE% of the nanovesicles was positively impacted by the concentrations of PL (A) and ethanol (B), but negatively impacted by the concentration of glycerin (C) by a significant direction. Another significant interaction effect of factors A and B on the EE% was recorded (Figure S5). The quadratic terms of factors A and B demonstrated significant impacts on the R_3_ response as shown by their low *p* values, while the other terms were non-significant (Table S1).

#### 3.1.4. Optimization Technique

The numerical optimization method based on BBD was utilized to achieve an optimum formulation of nanovesicles while adhering to a predetermined set of constraints as outlined in Table 1. The optimal formulation for RS-loaded nanovesicles contained 359.77 mg PL, 20% ethanol, and 15.76% glycerin and had a desirability value of 0.822. It was then evaluated in terms of its performance in relation to the three different responses. The measured values for the observed parameters were 302.90 ± 9.41 nm, −37.25 ± 0.39 mV, and 73.03 ± 0.72%, respectively. These values were found to be comparable to the predicted values (319.67 nm, −39.25 mV, and 72.6%) for the optimized formulation. The results of this study would provide evidence for the effectiveness of the optimization process in developing formulations of RS-loaded nanovesicles utilizing the BBD.

### 3.2. Characterization of Optimized RS-Loaded Glycethosomal formulation

#### 3.2.1. TEM Study

**Figure 2:**
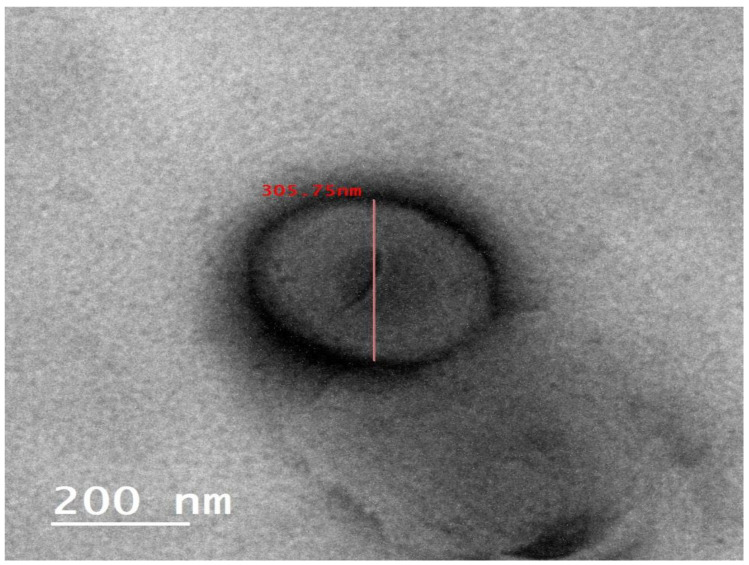
TEM image of optimized RS-loaded glycethosomal formulation.

The structure, size, and shape of the optimized RS-loaded glycethosomal formulation were investigated using TEM. The glycethosome was summarized into spherical vesicles with definite walls as shown in Figure 2. Additionally, TEM was used to quantify the VS of the optimized glycethosome. The VS obtained from dynamic light scattering method was equivalent to the result estimated by TEM.

#### 3.2.2. DSC Study

**Figure 3:**
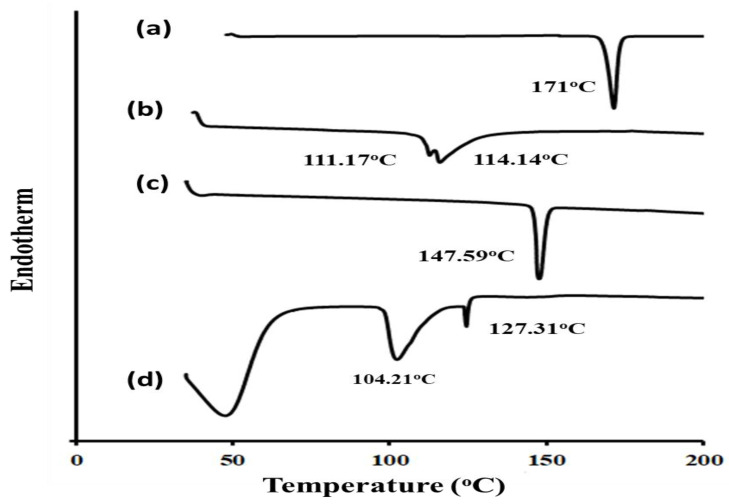
DSC thermograms of (a) pure RS, (b) PL, (c) cholesterol, and (d) optimized RS-loaded glycethosomal formulation.

To investigate the alterations in the drug crystallinity after loading in the optimized glycethosomal formulation, the DSC investigation was conducted (Figure 3). The thermogram of the pure RS sample displayed a distinct endothermic peak at a temperature of 171 °C. This peak was indicative of the melting point of RS and suggested its crystalline characteristics. The thermal behavior of PL and cholesterol showed specific endothermic peaks as shown in Figure 3.

Of interest, the DSC peaks of PL and cholesterol displayed shifts to lower temperatures. This might be ascribed to the possible solubilization of the lipid phase in the mixture of ethanol and glycerin used for the preparation of the glycethosomal formulation [9]. It was noteworthy that the thermogram of the optimized glycethosomal formulation loaded with RS did not display the expected endothermic peak typically observed for pure RS. This observation could suggest that the RS molecules were either dispersed inside the glycethosomes or that the solubility of RS had been enhanced in the manufactured glycethosomes.

#### 3.2.3. FTIR Study

**Figure 4:**
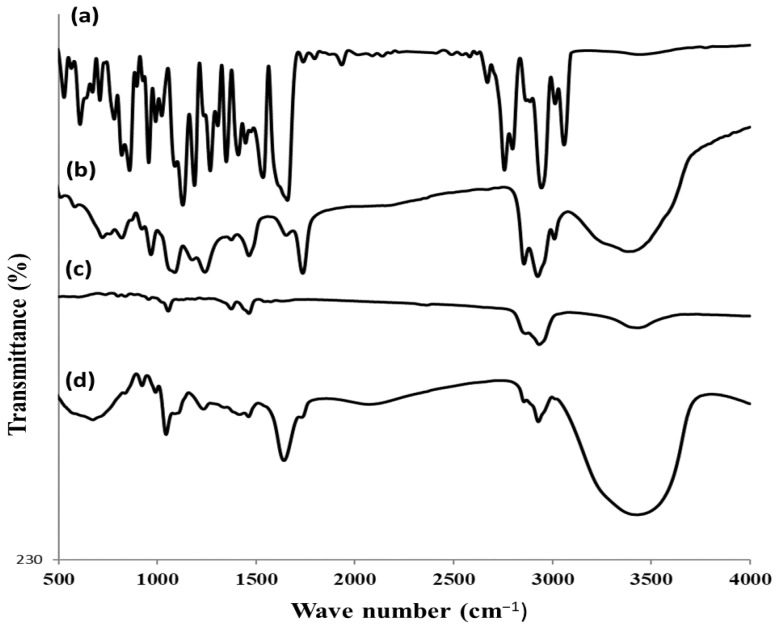
FTIR spectra of (a) pure RS, (b) PL, (c) cholesterol, and (d) optimized RS-loaded glycethosomal formulation.

To investigate the compatibility of RS and excipients used, the FTIR investigation was conducted (Figure 4). The spectrum of pure RS sample displayed several distinguishing absorption bands at 3060 cm^−1^ for the aromatic C–H group, 2946 and 2758 cm^−1^ for the aliphatic C–H group, 1660 cm^−1^ for the C=O group, 1536 cm^−1^ for the aromatic C=C group, 1448 cm^−1^ for the C=N group, 1411 cm^−1^ for the aliphatic C–H bending, 1350 cm^−1^ for the C–N group, 1130 cm^−1^ for the C–F group, and 860 cm^−1^ for the aromatic C–H bending group. The FTIR spectrum of PL showed characteristic C–H stretching bands of long fatty acid chain at 2925 and 2856 cm^−1^. It also showed stretching bands of C=CH, C=O, C–O, P–O–C, and N^+^(CH_3_)_3_ at 3009, 1738, 1246, 1093, and 969 cm^−1^, respectively. The FTIR spectrum of cholesterol showed broad bands at 3433 cm^−1^ related to the O–H group and 2933 and 2879 cm^−1^ related to the aliphatic C–H group. The band at 1461 cm^−1^ was due to the asymmetric stretching vibrations of the CH_2_ and CH_3_ groups. The band at 1374 cm^−1^ was ascribed to the CH_2_ and CH_3_ bending vibrations of cholesterol molecule.

The FTIR spectrum of RS-loaded glycethosomal preparation manifested that some weak interactions between RS and lipid constituents were present, which might be due to the formation of H-bonds or other weak bonds, such as van der Waals forces as shown by the broad peak between 3700–3000 cm^−1^ and the shifted C=O peak of PL (to 1641 cm^−1^). Those interactions could support the formation of the vesicular shapes and stabilization of their structures as reported by Sambhakar et al. [44]. Additionally, the disappearance of RS characteristic peaks, which were covered by those of PL and cholesterol, emphasized the entry of RS into the cavity of glycethosomal vesicles having a favorable encapsulation capacity [34].

#### 3.2.4. In Vitro Release Study

**Figure 5:**
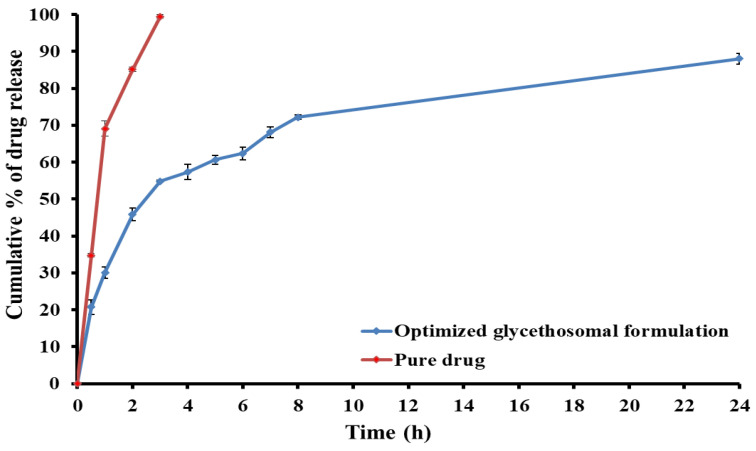
In vitro release of RS from optimized RS-loaded glycethosomal formulation.

Figure 5 depicts the in vitro release of RS from the optimized glycethosomal formulation. The pure drug exhibited a significantly elevated rate of drug release (*p* < 0.05), with almost 99% of the drug being released within 3 h. In contrast, after 3 h, 54.8% of RS was released from the optimized RS-loaded glycethosome. Furthermore, the optimized glycethosomal formulation exhibited a biphasic release profile that was defined by a rapid initial drug release in the first 3 h and a continuous release pattern for the next hours. Moreover, it was observed that the release of RS from the glycethosomal formulation was sustained over 24 h and this would confirm the ability of glycethosomes to entrap RS and retard its release.

### 3.3. Development of RS-Loaded Glycethosomal In Situ Gel Formulations

Concerning the preliminary study for preparing the in situ gel formulations, different concentrations of P407 were firstly examined. P407 could have the capability for forming micelles that could pack together in a highly ordered structure lattice acting as a driving force for gel formation [45]. Table 3 shows that there was an inverse correlation noted between the concentration of the polymer and the T_sol-gel_ value. P407 of 20% *w*/*w* concentration was selected for further investigations based on its T_sol-gel_ value close to 34 °C similar to the human nasal temperature. The obtained results were consistent with the findings of Abdulla et al. [45].

Different mucoadhesive agents (carbopol 940, HPMC-K4M, or PVP K30) at different concentrations were added to the optimized RS-loaded glycethosomal formulation at different gel/liquid glycethosome ratio (Table 4) to help the formulation to remain adhered to the mucosal membrane [46]. The optimization was based according to the gelation time (accepted value = close to 10 sec at a constant T_sol-gel_ of 34 °C) and the gel strength (accepted value = 25–50 sec). The formulations (G2, G5, and G9) that show acceptable gelation time and gel strength were selected for further characterization (Table 5).

### 3.4. Characterization of RS-Loaded Glycethosomal In Situ Gel Formulations

#### 3.4.1. Determination of pH

The values of pH of various in situ gel preparations are summarized in Table 5. The pH values were in the range of the nasal physiologic pH. This pH would encourage the non-irritant adhesion of the gel formulations to the nasal mucosa and result in a prolonged effect with an intermediate permeation release rate [47].

#### 3.4.2. Viscosity Determination

The significance of viscosity is crucial in the optimization of intranasal formulations as it governs their flow characteristics, spreadability, drug release, and duration of residence on the nasal mucosa [48]. Viscosity values obtained for different in situ preparations are presented in Table 5. The developed in situ gel formulation (G5) had a lower viscosity value (11,133.33 ± 47.14 cP) in comparison to the other studied gel formulations.

#### 3.4.3. Spreadability Determination

Spreadability is a characteristic parameter that pertains to the capacity of the gel to disperse and cover a specific surface area when administered onto the nasal mucosa. It is an important aspect of the intranasal formulation that enables uniform gel application and drug delivery at the right dose [49]. It was noted that gel spreadability was inversely proportional with the viscosity of various in situ gel preparations (Table 5). The produced G5 formulation exhibited high spreadability (3.26 ± 0.05 cm) and low viscosity (11,133.33 ± 47.14 cP) compared to G2 and G9. Similar findings were presented by Morsy et al., 2019 [50].

#### 3.4.4. Mucoadhesive Strength Study

The mucoadhesive strength values of G2, G5, and G9 formulations were calculated as represented in Table 5. In accordance to the findings of Wang et al. [51], it was determined that intranasal administration could be considered acceptable when the mucoadhesive strength values fall within the range of 4000–6000 dynes/cm^2^. According to Chaudhary and Verma [52], the presence of enough mucoadhesive strength could improve the residence time of formulations within the nasal cavity. This could effectively limit their removal and hence extend the release of drugs. Conversely, an excessive level of mucoadhesive strength would have the potential to cause injury to the mucosal membrane. Hence, it was important to determine the optimal mucoadhesive strength values of the intranasal gels.

#### 3.4.5. In Vitro Release Study

**Figure 6:**
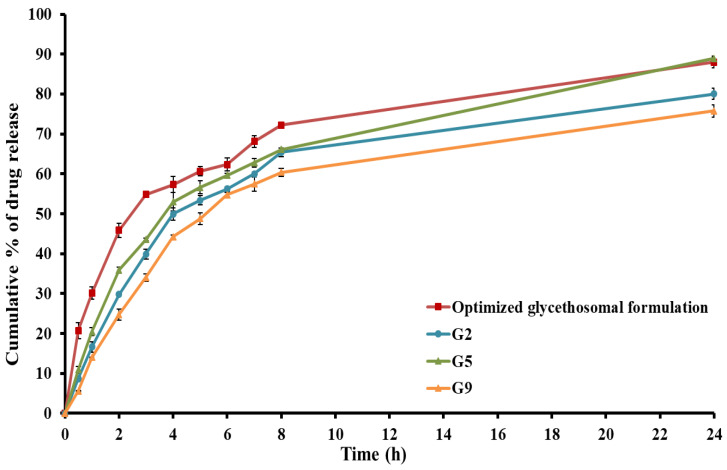
In vitro drug release from different RS-loaded glycethosomal in situ gel formulations.

Figure 6 illustrates the release profile of RS from the glycethosomal in situ gel formulations compared to their corresponding optimized liquid glycethosomal formulation. A slower in vitro release rate of RS was observed from glycethosomal-based in situ gels and this could be attributed to the increased viscosity resulting from the formation of three-dimensional gel networks [53]. Furthermore, the reduced rate of RS release observed from the in situ gel formulations could be related to the presence of glycethosomal vesicles that could encapsulate the drug. These vesicles were coated with a polymeric surface, which necessitated a longer path for the drug to traverse before being released into the surrounding medium. As a result, the overall RS release is diminished. However, the inclusion of RS-loaded glycethosomes in gel bases did not have a negative impact on drug release. In fact, over 75% of the loaded RS was released from various gel formulations within 24 h. Therefore, from the point of application, a glycethosomal in situ gel would be preferred. After examining the aforementioned parameters, formulation (G5) showed acceptable pH, viscosity, spreadability, and mucoadhesive strength. Additionally, it displayed a drug release profile close to that of the optimized liquid glycethosome. Hence, formulation (G5) was selected as an optimized RS-loaded glycethosomal in situ gel formulation and investigated for ex vivo permeation study, histopathology, and in vivo evaluations.

### 3.5. Characterization of Optimized RS-Loaded Glycethosomal In Situ Gel formulation

#### 3.5.1. Ex Vivo Permeability Study

**Figure 7:**
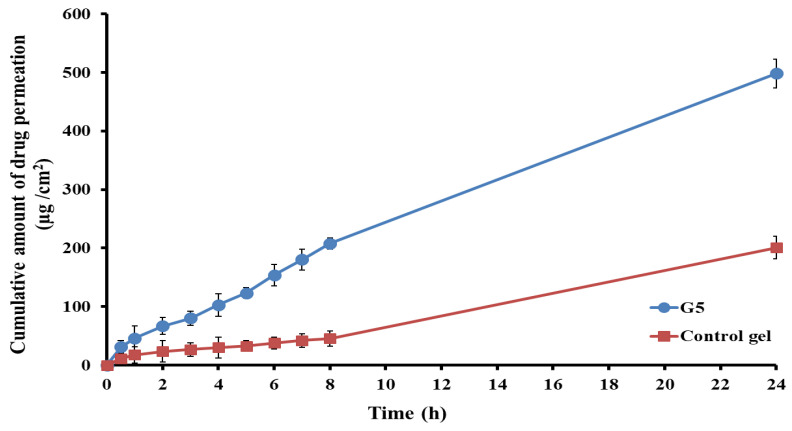
Ex vivo permeation of RS from control in situ gel and optimized glycethosomal in situ gel formulation (G5).

The ex vivo RS permeation from the optimized glycethosomal in situ gel or control in situ gel across the nasal mucosa was investigated as a marker for predicting the overall in vivo performance. The results presented in Figure 7 demonstrate that the incorporation of RS into the glycethosomal in situ gel (G5) led to a significant enhancement in the penetration of RS through the nasal mucosa as compared to the control in situ gel (comprised of the same gel composition of G5 loaded with pure RS powder). This difference was found to be statistically significant (*p* < 0.001). After 24 h, the optimized RS-loaded glycethosomal in situ gel showed a cumulative drug permeation of 498.34 ± 24.67 μg/cm^2^ through the nasal mucosa compared to the RS-loaded control in situ gel, which exhibited a cumulative drug permeation of 200.76 ± 19.53 μg/cm^2^. The significant difference between the optimized gel and control gel formulations could be observed in Table 6 where the former displayed substantially higher J_ss_ and K_p_ values than those of the latter. This would help the potential of optimized gel to greatly enhance the permeation of RS through the nasal membrane (E_r_ value of 5.43) and promote its penetration performance.

The enhanced permeability of the optimized RS-loaded glycethosomal in situ gel might be attributed to the formulation’s ingredients to compromise the lipid surface barrier of the nasal mucosa. Furthermore, the appropriate viscosity of the optimum preparation might influence the absorption of the drug through the nasal mucosa [54]. Additionally, the action of ethanol in fluidizing the lipid bilayer in the nasal mucosa and softening the vesicles could help them to squeeze through the deeper mucosal layer [34]. The moisturizing and penetration promoting action of glycerol would facilitate the delivery of drugs through the nasal mucosa [55]. Hence, the dual actions of ethanol and glycerol could help the glycethosomal gel formulation to be a drug reservoir that could efficiently transport RS through the nasal mucosa.

#### 3.5.2. Histopathological Study

**Figure 8:**
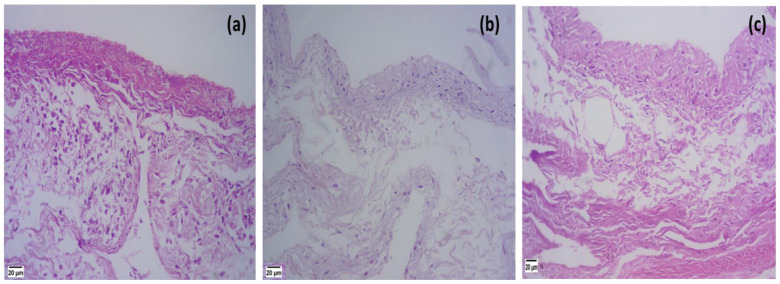
Histopathological study of sheep nasal mucosa treated with (**a**) saline, (**b**) isopropyl alcohol, and (**c**) optimized RS-loaded glycethosomal in situ gel formulation (G5).

A histopathological examination was conducted to explore the potential harmful effects of the components of the glycethosomal in situ gel formulation (G5) loaded with RS on the nasal mucosa [56]. The histopathological study demonstrated that the epithelial layers of the mucosal sections treated with saline solution remained undamaged showing no evidence of nasociliary injury or inflammation, such as erythema or edema (Figure 8a). The nasal cilia of the parts treated with isopropyl alcohol exhibited significant destruction and widespread damage to the mucosal tissue as depicted in Figure 8b. In relation to the optimized RS-loaded glycethosomal in situ gel formulation, it was observed that the epithelial layers remained undamaged and exhibited no indications of toxicity (Figure 8c). These findings would confirm the safety and bio-compatibility of the components under investigation [26].

#### 3.5.3. In Vivo Pharmacokinetic Study

**Figure 9:**
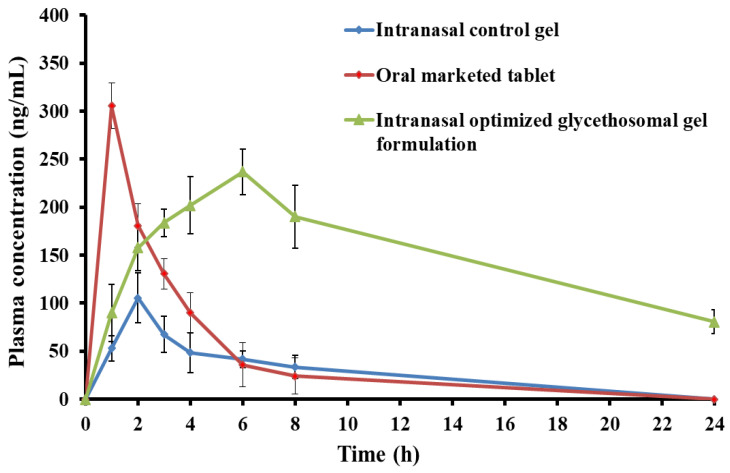
Plasma concentration-time curve of RS after administration of optimized RS-loaded glycethosomal In situ gel formulation, control in situ gel, and oral marketed tablet into rats.

Figure 9 depicts the blood concentration-time profile of RS subsequent to the intranasal administration of the optimum formulation (G5) in comparison to the intranasal control gel and the oral marketed tablet. The results clearly indicated that the optimized formulation exhibited much greater concentrations of RS in plasma compared to the control RS gel. The C_max_ of RS after intranasal administration of the optimum gel preparation was found to be 236.87 ± 23.67 ng/mL, which was substantially greater than the C_max_ observed after application of the control gel (105.54 ± 25.74 ng/mL) (Table 7). The RS-loaded glycethosomal in situ gel formulation showed a t_max_ value of 6 h, which was notably longer compared to the t_max_ values of 2 h for the control gel and 1 h for the orally administered marketed tablets (Table 7). The observed increase in the t_max_ value of the optimized intranasal gel formulation (*p* < 0.05) relative to the other formulations under investigation would suggest the potential controlled release mechanism of the glycethosomal gel system. According to the findings of Said and Elmenoufy [57], it was seen that the utilization of sustained release formulations resulted in a reduction of adverse effects, as well as an enhancement in the efficacy and bioavailability of the drug.

Furthermore, the AUC_0-t_ for the optimized RS-loaded glycethosomal gel formulation was 3610.84 ± 69.38 ng/mL^−1^·h. This value was significantly greater than the control in situ gel (442.74 ± 36.53 ng/mL^−1^·h) and the oral marketed formulation (1106.39 ± 55.14) (*p* < 0.05). This could indicate the prolonged RS release after the intranasal application of glycethosomal gel systems resulting in higher relative bioavailability of RS when compared to the control gel and oral marketed tablets.

Moreover, the t_1/2_ was significantly increased from 1.88 ± 0.61 h for the oral marketed tablet and 7.52 ± 0.33 h for the control gel to 12.08 ± 0.87 h for the optimized glycethosomal gel formulation. These findings expressed the sustained release of RS from the optimized glycethosomal gel formulation and the drug existence in the systemic circulation for an extended duration in comparison to the control gel and the oral marketed tablet [29].

The optimized RS-loaded glycethosomal gel preparation efficiently prolonged the MRT of RS in the systemic circulation. The MRT of the optimized formulation compared to the control gel and the oral market tablet were 18.44 ± 0.96 h, 10.41 ± 0.81 h, and 2.50 ± 0.14 h, respectively. Interestingly, incorporation of RS into glycethosomal gel resulted in a significant enhancement of the RS systemic bioavailability. The optimized formulation showed an eight-fold increase in the relative bioavailability compared to that of the control gel and three times higher than that of the oral marketed tablet. The findings could suggest that the systemic bioavailability of RS could be significantly increased when being incorporated in the glycethosomal gels in comparison to the other tested preparations.

#### 3.5.4. Effect of Optimized Gel Formulation on Ketamine-Induced Hippocampal Neurotransmitters’ Content, Oxidative Stress, Inflammation, and BDNF

Schizophrenia is a chronic disease that results from disturbances in the brain neurotransmitter levels. Ketamine was reported to be an N-methyl-D-aspartate (NMDA) receptor antagonist that was commonly utilized to produce schizophrenia-like symptoms in animals after chronic intraperitoneal injection [58].

**Figure 10:**
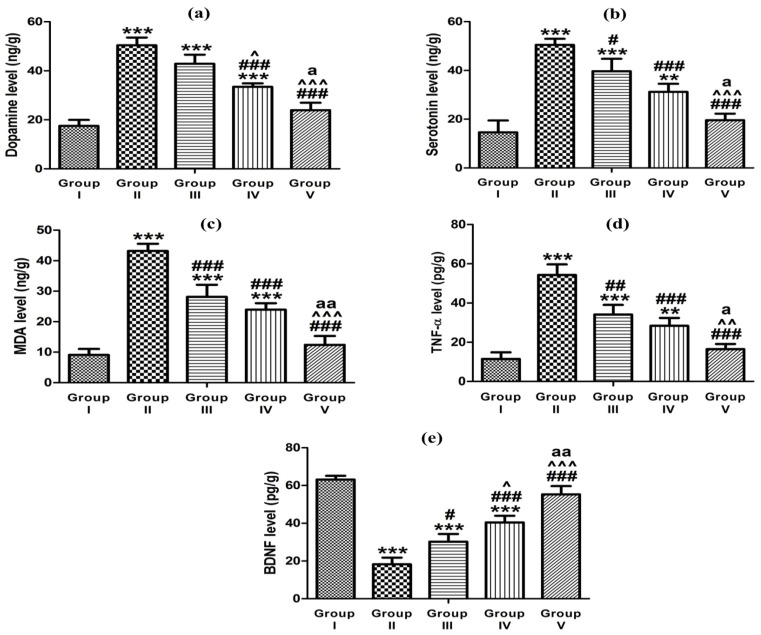
Effect of studied formulations on ketamine-induced hippocampal (**a**) dopamine level, (**b**) serotonin level, (**c**) MDA level, (**d**) TNF-α level, and (**e**) BDNF level. In comparison to group I: ** *p* < 0.01, *** *p* < 0.001. In comparison to group II: ^#^ *p* < 0.05, ^##^ *p* < 0.01, ^###^ *p* < 0.001. In comparison to group III: ^^^ *p* < 0.05, ^^^^ *p* < 0.01, ^^^^^ *p* < 0.001. In comparison to group IV: ^a^ *p* < 0.05, ^aa^ *p* < 0.01.

In ketamine-induced schizophrenic rats, the levels of dopamine and serotonin neurotransmitters in the hippocampus were significantly higher than the control value by 2.9 and 3.6 folds, respectively (Table 8 and Figure 10 a,b). The high hippocampal dopamine and serotonin contents following ketamine injection could imply the substantial role of these neurotransmitters in the pathophysiology of schizophrenia [59]. The administration of the control in situ gel (group III), oral market preparation (group IV), and glycethosomal in situ gel (group V) effectively reduced the increased dopamine levels by 1.2, 1.5, and 2.1 folds, respectively, and effectively reduced the increased serotonin values by 1.3, 1.6, and 2.6 folds, respectively, in comparison to the ketamine-induced schizophrenic animal (group II). Ibrahim et al. [29] reported the affinity of RS to the D_2_ receptor and its higher affinity to the 5-HT_2A_ receptor. Our results demonstrated that RS-loaded glycethosomal in situ gel could efficiently reverse the abnormalities in neurotransmitters caused by ketamine and this would confirm the neuroprotective RS properties.

Oxidative stress has a crucial role in the neuronal injury and pathophysiology of schizophrenia [60]. The administration of ketamine into rats resulted in a considerable increase in the MDA levels inside the hippocampus reaching a 4.7-fold elevation compared to the control group (Table 8 and Figure 10 c). The NMDA receptor antagonism by ketamine could lead to a cascade of excitotoxic events, such as oxidative stress and apoptosis [30]. The administration of control gel, oral marketed preparation, and optimized gel to the schizophrenic rats resulted in a considerable reduction in the MDA levels. Specifically, the MDA levels were lowered by 1.5, 1.8, and 3.5 folds, respectively, compared to the schizophrenic rats (group II). These findings represented the potential of optimized glycethosomal in situ gel to restore the oxidative balance. Hendouei et al. [61] reported that the desirable anti-oxidant activity of the drug might be involved in its anti-psychotic action.

Similarly, there was a considerable increase in the TNF-α level in the hippocampus following the ketamine therapy with levels reaching 4.7-fold in comparison to the control animals (Table 8 and Figure 10 d). Stress caused by ketamine could promote the production of proinflammatory cytokines and their release, such as TNF-α causing a disruption in the brain neurotransmitter system [62]. The administration of studied formulations resulted in a significant reduction in the high TNF-α levels by 1.6, 1.9, and 3.3 times for the control gel, market tablet, and optimized glycethosomal gel, respectively, when compared to animals injected with ketamine (group II). The elevation in the proinflammatory cytokine (TNF-α) was significantly ameliorated expressing the remarkable anti-inflammatory effectiveness and the neuroprotective action of RS when being loaded in the optimized glycethosomal in situ gel.

Ketamine significantly reduced the BDNF level in the hippocampus exhibiting a 3.5-fold reduction compared to the control animals (group I) (Table 8 and Figure 10e). The minimized levels of hippocampal BDNF caused by ketamine could result in synaptic plasticity loss as a possible mechanism for inducing schizophrenia [63]. The administration of formulations given to the animal groups III, IV, and V resulted in a considerable increase in the BDNF level by 1.7, 2.2, and 3.1 times, respectively, compared to rats treated with ketamine (group II). The marked action of the optimized formulation in comparison to the other studied preparations could indicate its potentiality for enhancing the RS permeability through the nasal mucosa and reaching the brain in a superior manner. Eventually, the incorporation of RS in glycethosomal in situ gels could help reduce the frequency of RS dosing, minimize the side effects occurrence, and improve the effectiveness and bioavailability of the drug.

## 4. Conclusions

In our investigation, the anti-psychotic drug, RS, was successfully introduced in a glycethosomal in situ gel system. The formulated RS-loaded glycethosomal systems were optimized using the BBD. The optimized formulation for RS-loaded nanovesicles, obtained at a desirability value of 0.822, was composed of 359.77 mg PL, 20% ethanol, and 15.76% glycerin. The optimized glycethosomal system showed smooth sphere-shaped nanosized vesicles with a reasonable ZP and sustained in vitro RS release. After incorporation into in situ gel bases, the prepared glycethosomal gel formulations exhibited acceptable physical properties in terms of pH, viscosity, spreadability as well as considerably boosted the nasal permeability of RS across the nasal mucosa. Significantly, in vivo pharmacokinetic investigations have demonstrated the effectiveness of the optimum glycethosomal in situ gel formulation in enhancing the systemic bioavailability of RS. This enhancement is evidenced by an eight-fold increase in AUC_0-t_ when compared to the in situ control gel and a three-fold increase when compared to the oral marketed tablet. The modulation of brain neurotransmitters in addition to the anti-oxidant and anti-inflammatory action of RS were desirably enhanced following the incorporation of RS in the glycethosomal in situ gels in comparison to the control in situ gel and the oral marketed tablet. To sum up, glycethosomal in situ gel might be a prominent strategy for enhancing the intranasal delivery of RS and augmenting its efficacy in the management of schizophrenia.

## Figures and Tables

**Table 1:** BBD for optimization of RS-loaded lipid-based nanovesicles.

Independent Variables	Symbol	Level of Variation	
		Low	High
PL amount (mg)	A	200	400
Ethanol concentration (% *v*/*v*)	B	0	20
Glycerin concentration (% *v*/*v*)	C	0	20
**Dependent variables**	**Symbol**	**Constraint**	
VS (nm)	R_1_	Minimize	
ZP (mV)	R_2_	Maximize	
EE% (%)	R_3_	Maximize	

PL, phospholipid; VS, vesicle size; ZP, zeta potential; EE%, entrapment efficiency.

**Table 2:** Composition of different RS-loaded lipid-based nanovesicles with their responses using BBD.

Type of Formulation	Run ^(e)^	A: PL Amount (mg)	B: Ethanol Concentration (% *v*/*v*)	C: Glycerin Concentration (% *v*/*v*)	R_1_: VS (nm)	R_2_: ZP (mV)	R_3_: EE% (%)
Glycerosome ^(a)^	F1	400	0	10	646.80 ± 31.65	25.30 ± 1.95	57.86 ± 0.87
Glycethosome ^(b)^	F2	300	20	20	231.30 ± 19.18	39.25 ± 0.42	67.20 ± 1.15
Glycethosome	F3	400	20	10	410.50 ± 14.08	37.86 ± 0.99	78.16 ± 1.68
Glycethosome	F4	200	10	20	328.90 ± 20.11	27.76 ± 1.51	55.37 ± 0.99
Glycethosome	F5	300	10	10	292.30 ± 12.63	26.66 ± 1.05	62.29 ± 1.05
Ethosome ^(c)^	F6	400	10	0	352.20 ± 11.61	26.16 ± 1.10	70.30 ± 1.81
Glycethosome	F7	300	10	10	259.20 ± 15.94	26.76 ± 0.94	61.23 ± 0.85
Glycerosome	F8	300	0	20	571.70 ± 26.81	27.91 ± 0.95	47.62 ± 1.65
Glycethosome	F9	300	10	10	276.20 ± 10.13	28.15 ± 1.22	62.01 ± 1.12
Glycethosome	F10	200	20	10	163.10 ± 7.45	28.56 ± 0.75	60.58 ± 0.57
Ethosome	F11	200	10	0	180.90 ± 16.73	19.57 ± 1.15	57.05 ± 1.10
Ethosome	F12	300	20	0	189.70 ± 20.19	28.66 ± 0.88	69.11 ± 1.41
Glycerosome	F13	200	0	10	473.70 ± 10.08	17.26 ± 0.65	47.22 ± 1.95
Glycethosome	F14	400	10	20	442.30 ± 16.88	38.58 ± 0.81	65.23 ± 1.81
Liposome ^(d)^	F15	300	0	0	466.50 ± 29.15	15.92 ± 1.31	50.83 ± 0.89
Glycethosome	F16	300	10	10	278.50 ± 18.05	25.81 ± 0.91	60.45 ± 1.25
Glycethosome	F17	300	10	10	296.50 ± 9.93	28.25 ± 1.05	60.99 ± 1.31

PL, phospholipid; VS, vesicle size; ZP, zeta potential; EE%, entrapment efficiency; ^(a)^, glycerosomal formulations contained no ethanol; ^(b)^, glycethosomal formulations contained both ethanol and glycerin; ^(c)^, ethosomal formulations contained no glycerin; ^(d)^, liposomal formulations contained no ethanol or glycerin; ^(e)^, all formulations contained a constant concentration of PG at 5% *v*/*v*.

**Table 3:** Determination of T_sol-gel_ values of different P407 polymeric solutions at different concentrations.

Gel Base	T_sol-gel_ (°C)
P407 (17% *w*/*w*)	>60
P407 (18% *w*/*w*)	44.67 ± 0.47
P407 (19% *w*/*w*)	37.00 ± 0.82
P407 (20% *w*/*w*)	33.67 ± 0.49

P407, poloxamer 407; T_sol-gel_, solution-to-gel temperature.

**Table 4:** Composition and evaluation of different RS-loaded glycethosomal in situ gel formulations.

Formulation	Gel Base	Mucoadhesive Gent	Gel/Glycethosome Ratio (*w*/*w*)	Gelation Time (sec)	Gel Strength (sec)
G1	P407 (20% *w*/*w*)	Carbopol 940 (0.1% *w*/*w*)	4:1	No gelation	-
			5:1	No gelation	-
			6:1	30.28 ± 0.66	22.34 ± 0.85
**G2**	**P407** **(20% *w*/*w*)**	**Carbopol 940** **(0.2% *w*/*w*)**	2:1	No gelation	-
			3:1	35.47 ± 0.92	18.30 ± 1.12
			**4:1**	**20.12 ± 0.15**	**29.23 ± 1.01**
G3	P407 (20% *w*/*w*)	Carbopol 940 (0.3% *w*/*w*)	2:1	No gelation	-
			3:1	44.37 ± 1.02	23.25 ± 0.91
			4:1	10.80 ± 0.79	> 50
G4	P407 (20% *w*/*w*)	HPMC-K4M (0.7% *w*/*w*)	4:1	No gelation	-
			5:1	36.73 ± 0.84	23.27 ± 0.94
			6:1	24.35 ± 1.12	27.40 ± 0.34
**G5**	**P407** **(20% *w*/*w*)**	**HPMC-K4M** **(1% *w*/*w*)**	2:1	No gelation	-
			3:1	No gelation	-
			**4:1**	**12.36 ± 0.19**	**32.74 ± 0.64**
G6	P407 (20% *w*/*w*)	HPMC-K4M (1.2% *w*/*w*)	2:1	No gelation	-
			3:1	44.37 ± 1.02	39.84 ± 0.43
			4:1	10.80 ± 0.79	> 50
G7	P407 (20% *w*/*w*)	PVP K30 (0.5% *w*/*w*)	4:1	No gelation	-
			5:1	38.45 ± 0.59	12.92 ± 1.23
			6:1	27.01 ± 0.93	15.75 ± 0.78
G8	P407 (20% *w*/*w*)	PVP K30 (0.7% *w*/*w*)	4:1	No gelation	-
			5:1	36.28 ± 0.73	16.19 ± 0.18
			6:1	20.63 ± 1.93	20.13 ± 0.90
**G9**	**P407** **(20% *w*/*w*)**	**PVP K30** **(1% *w*/*w*)**	3:1	No gelation	-
			4:1	34.23 ± 0.27	30.10 ± 0.19
			**5:1**	**10.38 ± 0.91**	**35.27 ± 1.10**

P407, poloxamer 407; HPMC-K4M, hydroxypropyl methyl cellulose-K4M; PVP K30, polyvinylpyrrolidone K30.

**Table 5:** Characterization of different RS-loaded glycethosomal in situ gel formulations.

Formulation	Gel Base	Mucoadhesive Agent	Gel/Glycethosome Ratio (*w*/*w*)	pH	Viscosity (cP)	Spreadability (cm)	Mucoadhesive Strength (dyne/cm^2^)
G2	P407 (20% *w*/*w*)	Carbopol 940 (0.2% *w*/*w*)	4:1	6.41 ± 0.04	15,366.67 ± 169.97	3.00 ± 0.08	4799.04 ± 63.71
G5	P407 (20% *w*/*w*)	HPMC-K4M (1% *w*/*w*)	4:1	6.58 ± 0.12	11,133.33 ± 47.14	3.26 ± 0.05	4163.23 ± 38.97
G9	P407 (20% *w*/*w*)	PVP K30 (1% *w*/*w*)	5:1	6.74 ± 0.02	16,366.67 ± 309.12	2.53 ± 0.12	5197.24 ± 53.05

P407, poloxamer 407; HPMC-K4M, hydroxypropyl methyl cellulose-K4M; PVP K30, polyvinylpyrrolidone K30.

**Table 6:** Ex vivo permeation studies of RS from control in situ gel and optimized glycethosomal in situ gel formulation (G5).

Permeation Parameter	Optimized Glycethosomal Gel (G5)	Control Gel
J_ss_ (μg/cm^2^·h^−1^)	22.11 ± 3.37	4.07 ± 1.12
K_p_ × 10^−3^ (cm/h)	11.06 ± 1.75	2.04 ± 0.58
E_r_	5.43	-

J_ss_, drug flux at steady state; K_p_, permeability coefficient; E_r_, enhancement ratio.

**Table 7:** Pharmacokinetic parameters of RS following administration of control in situ gel, oral marketed tablet, and optimized RS-loaded glycethosomal in situ gel formulation into rats.

Parameters	Control Gel	Oral Marketed Tablet	Optimized Glycethosomal Gel
C_max_ (ng/mL)	105.54 ± 25.74	305.51 ± 23.85	236.87 ± 23.67
t_max_ (h)	2	1	6
K_el_ (h^−1^)	0.092 ± 0.02	0.37 ± 0.12	0.057 ± 0.01
t_1/2_ (h)	7.52 ± 0.33	1.88 ± 0.61	12.08 ± 0.87
AUC_0-t_ (ng·mL^−1^·h)	442.74 ± 36.53	1106.39 ± 55.14	3610.84 ± 69.38
AUC_0-∞_ (ng·mL^−1^·h)	807.86 ± 40.79	1172.39 ± 48.71	5018.35 ± 53.46
AUMC_0-∞_ (ng·mL^−1^·h^2^)	8411.58 ± 73.24	2932.79 ± 64.41	92,519.05 ± 95.11
MRT (h)	10.41 ± 0.81	2.50 ± 0.14	18.44 ± 0.96
Relative bioavailability (%)	-	249.89	815.57

C_max_, maximum plasma concentration; t_max_, time for reaching this plasma concentration; K_el_, apparent elimination rate constant; t_1/2_, half-life; AUC_0-t_, area under the plasma concentration-time curve; AUC_0-∞_, area under the curve from time zero to infinity; AUMC_0-∞_, area under the first moment curve; MRT, mean residence time.

**Table 8:** Effect of studied formulations on ketamine-induced hippocampal neurotransmitters’ content, oxidative stress, inflammation, and BDNF.

Animal Groups	Dopamine (ng/g Tissue)	Serotonin (ng/g Tissue)	MDA (ng/g Tissue)	TNF-α (pg/g Tissue)	BDNF (pg/g Tissue)
Group I	17.52 ± 2.46	14.62 ± 4.83	9.10 ± 1.96	11.43 ± 3.42	63.19 ± 1.96
Group II	50.43 ± 3.15	50.46 ± 2.49	43.17 ± 2.39	54.27 ± 5.42	18.26 ± 3.51
Group III	42.86 ± 3.73	39.69 ± 5.10	28.15 ± 3.97	34.12 ± 4.90	30.20 ± 4.11
Group IV	33.49 ± 1.39	31.24 ± 3.26	23.95 ± 2.11	28.38 ± 4.01	40.41 ± 3.56
Group V	23.92 ± 3.06	19.57 ± 2.73	12.38 ± 2.93	16.45 ± 2.66	55.27 ± 4.42

MDA, malondialdehyde; TNF-α, tumor necrosis factor-α; BDNF, brain derived neurotrophic factor.

## Data Availability

Not applicable.

## Supplementary Materials

The following supporting information can be downloaded at: https://www.mdpi.com/article/10.3390/pharmaceutics15112521/s1, Table S1: Statistical analysis of three responses by ANOVA; Figure S1: Graphical representation of a modified balance for mucoadhesive strength measurement; Figure S2: Plots of predicted values versus actual values of (a) R_1_ response, (b) R_2_ response, (c) R_3_ response; Figure S3: Normal plots of residuals of (a) R_1_ response, (b) R_2_ response, (c) R_3_ response; Figure S4: Plots of externally studentized residuals versus runs of (a) R_1_ response, (b) R_2_ response, (c) R_3_ response; Figure S5: Interaction effect of factors A and B on R_3_ response. The black line indicates the low level of ethanol concentration and the red line indicated the high level of ethanol concentration.
