# Supplementary material for: Tailoring Risperidone-Loaded Glycethosomal In Situ Gels Using Box–Behnken Design for Treatment of Schizophrenia-Induced Rats via Intranasal Route
Source: Pharmaceutics. 2023 Oct 24;15(11):2521. doi: 10.3390/pharmaceutics15112521 (PMC10675145; doi:10.3390/pharmaceutics15112521)
Supplement: Supplementary file 1 [file pharmaceutics-15-02521-s001.zip › pharmaceutics-2640516-supplementary.pdf]

Supplementary Material

Table S1. Statistical analysis of three responses by ANOVA

Source	R_1 (quadratic model)		R_2 (linear model)		R_3 (quadratic model)	
	**p-value**	**Significance**	**p-value**	**Significance**	**p-value**	**Significance**
**Model**	< 0.0001	Significant	< 0.0001	Significant	< 0.0001	Significant
**A**	< 0.0001	Significant	< 0.0001	Significant	< 0.0001	Significant
**B**	< 0.0001	Significant	< 0.0001	Significant	< 0.0001	Significant
**C**	0.0013	Significant	< 0.0001	Significant	0.0058	Significant
**AB**	0.2004	Non-significant	-	-	0.0144	Significant
**AC**	0.3072	Non-significant	-	-	0.1583	Non-significant
**BC**	0.2656	Not-significant	-	-	0.5639	Non-significant
**A^2**	0.0048	Significant	-	-	0.0292	Significant
**B^2**	0.0002	Significant	-	-	0.0091	Significant
**C^2**	0.6225	Non-significant	-	-	0.1541	Non-significant
**Fit statistics**						
**R^2**	0.9842		0.9810		0.9922	
**Adjusted R^2**	0.9639		0.9766		0.9822	
**Predicted R^2**	0.7886		0.9678		0.9069	
**Adequate precision**	23.1811		46.9951		37.3075	

ANOVA, analysis of variance; A, PL amount; B, ethanol concentration; C, glycerin concentration; R_1, vesicle size (VS); R_2, zeta potential (ZP); R_3, entrapment efficiency (EE%); R^2, multiple correlation coefficient.

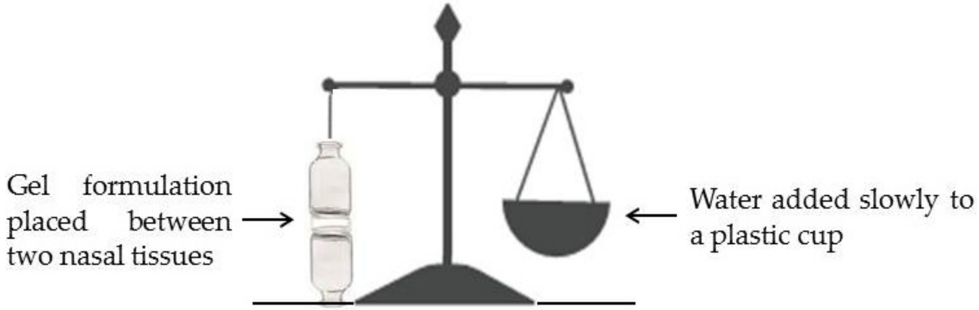

Figure S1. Graphical representation of a modified balance for mucoadhesive strength measurement.

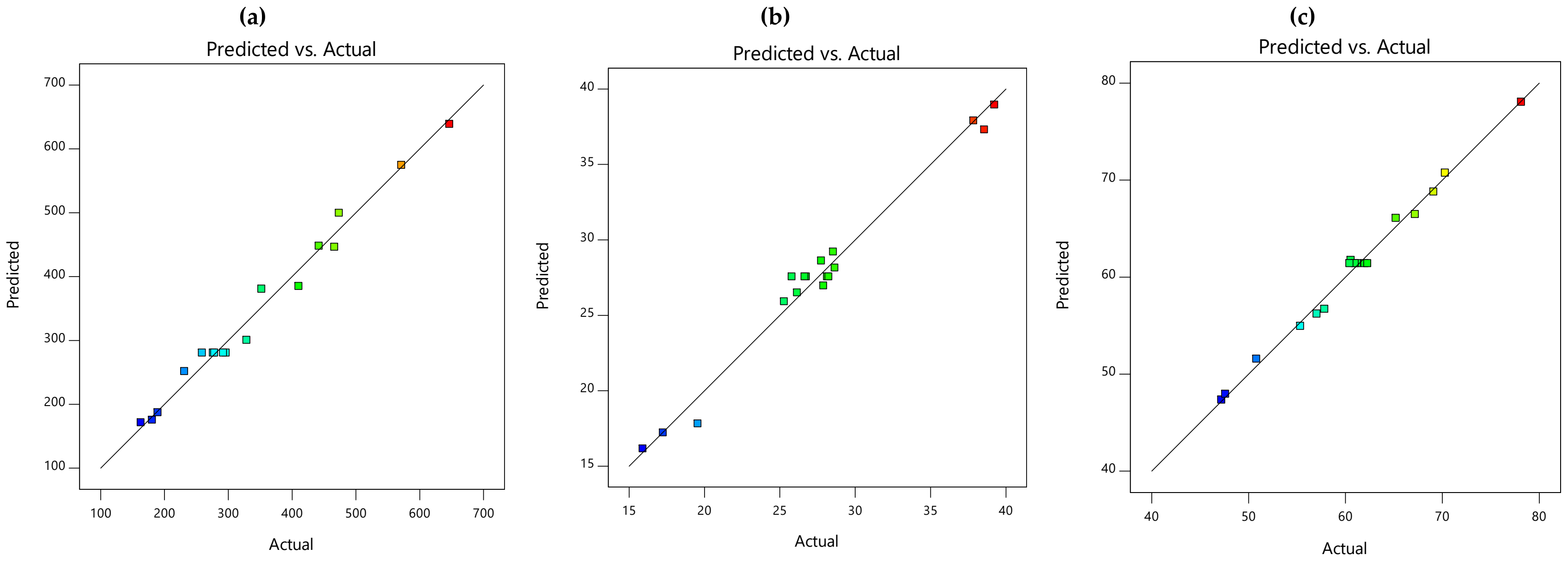

Figure S2. Plots of predicted values versus actual values of (a) R_1 response (b) R_2 response (c) R_3 response.

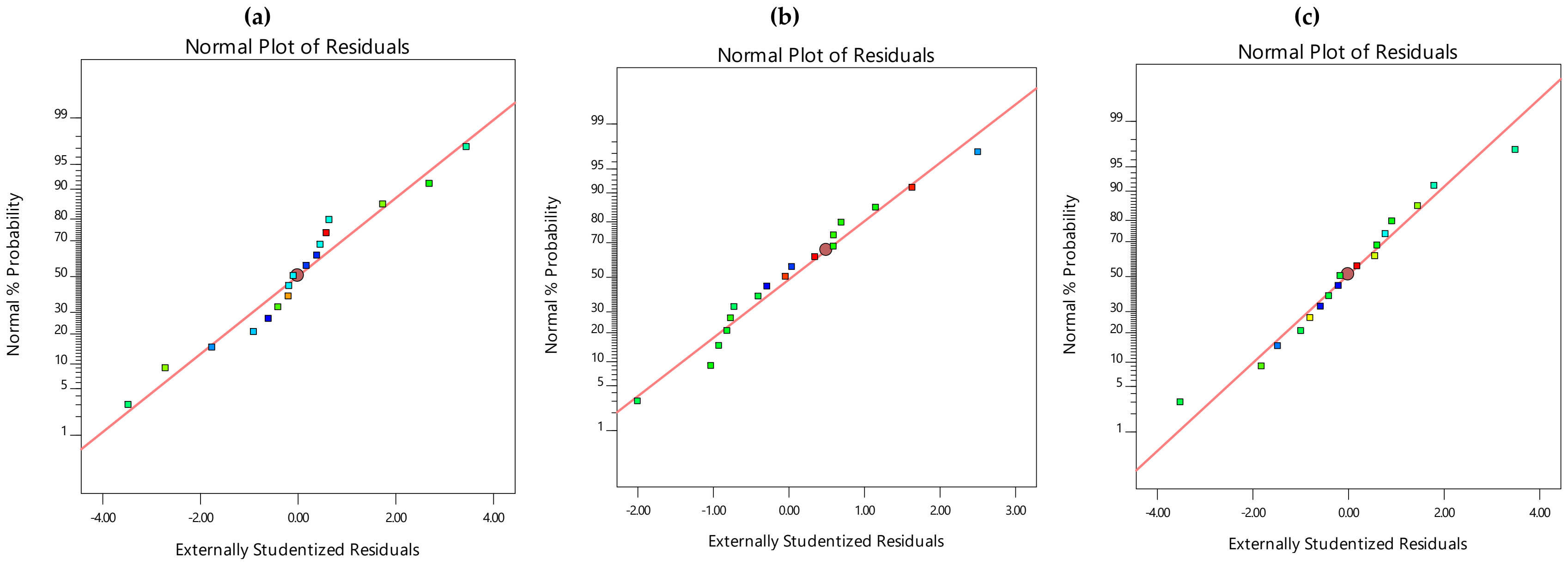

Figure S3. Normal plots of residuals of (a) R_1 response (b) R_2 response (c) R_3 response.

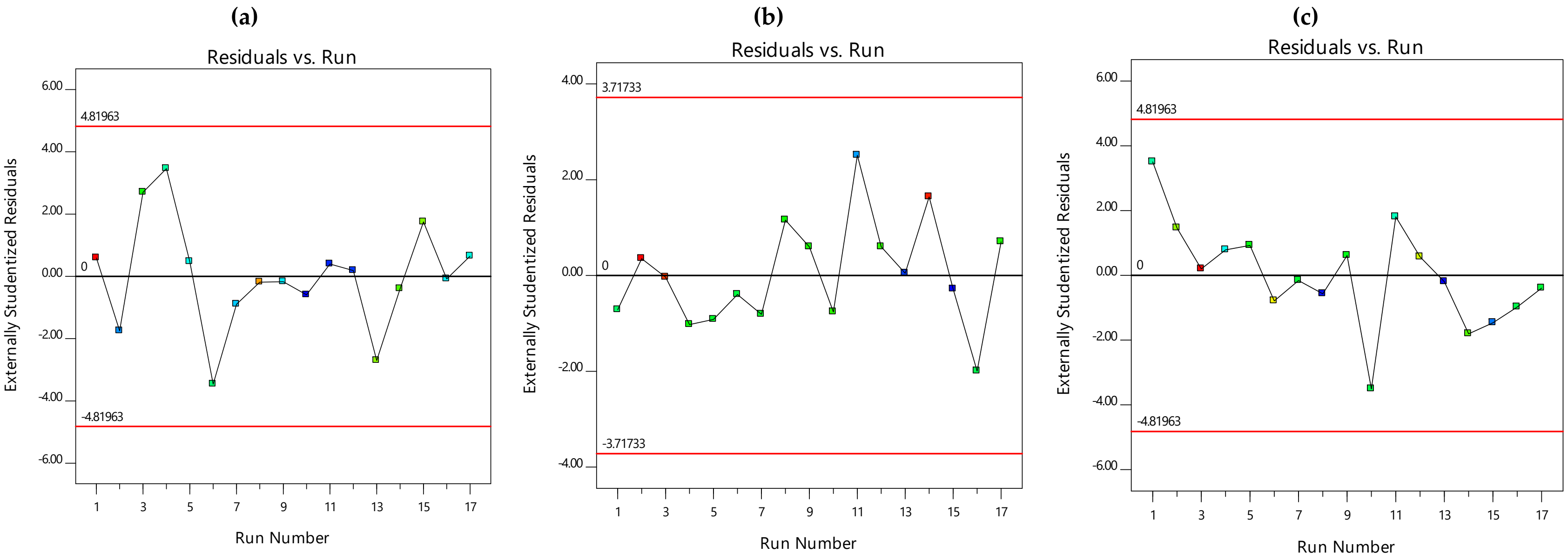

Figure S4. Plots of externally studentized residuals versus runs of (a) R_1 response (b) R_2 response (c) R_3 response.

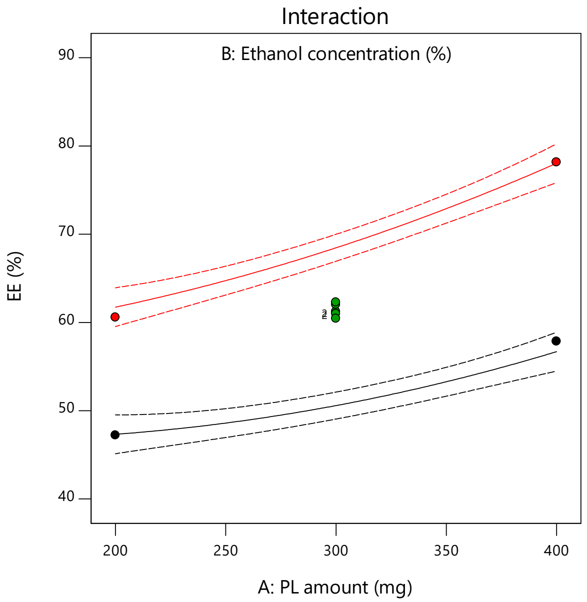

Figure S5. Interaction effect of factors A and B on R_3 response. The black line indicates the low level of ethanol concentration and the red line indicated the high level of ethanol concentration.

The interaction of independent factors by observing the effect of changing the levels of them on the response was studied. Non-parallelism of the two opposite lines inside the plot could demonstrate the presence of interaction between the two factors. It was seen that increasing the EE% of the vesicles was observed by increasing the PL concentration and using the lowest ethanol concentration (black line). However, higher rate of EE% increment was observed as induced by increasing the PL concentration and using higher ethanol concentration (red line). Hence, a significant interaction effect was found between the A and B factors on the R_3 response. It could be also supported by the data of Table S1 Supplementary Material showing the significant low p-value of AB interaction factor (0.0144).
